# Genetically engineered murine pancreatic cancer models approximate immunophenotypic properties of human patients

**DOI:** 10.1186/2051-1426-1-S1-P163

**Published:** 2013-11-07

**Authors:** Thomas A Mace, Jason Pitaressi, Reena Shakya, Wendy Frankel, Tim Eubank, Tanios Bekaii-Saab, Mark Bloomston, Mitch Phelps, Thomas Ludwig, Michael Ostrowski, Gregory B Lesinski

**Affiliations:** 1 The Ohio State University, Columbus, OH, USA

## 

Pancreatic ductal adenocarcinoma (PDA) has a prominent stroma which includes collagen, lymphoid, myeloid, and stellate cells. Adequate preclinical models are needed to advance immunotherapy for PDA. Indeed many xenograft models poorly mimic the morphology, stroma, and phenotype of the human PDA microenvironment. Thus, further development and use of relevant spontaneously arising genetically engineered murine models (GEMM) of PDA is important for accurate preclinical assessment of immune therapies. We have demonstrated that PDA-associated stellate cells promote the differentiation of myeloid-derived suppressor cells (MDSC) in a STAT3-dependent manner. Thus it is important that tumor and stroma in murine PDA models recapitulate a human patient’s immune phenotype. To date, the contribution of various cytokines or soluble factors in these models has not been well characterized. We hypothesized that various pancreatic cancer GEMM would accurately replicate alterations in STAT3 signaling and immune suppressive phenotypic properties both systemically and within the tumor microenvironment. We examined the role of IL-6/STAT3 signaling within the microenvironment from four separate spontaneously arising GEMM of PDA (KPC, BRCA1-KPC, BRCA2-KPC, and Mist1(KRasG12D/+) which recapitulate human tumors. KPC, BRCA-KPC, and Mist1(KRasG12D/+) mice develop spontaneously arising KRAS mutant (TP53 and BRCA2 mutant, respectively) pancreatic tumors. Similar to human PDA, fibrosis was evident in all tumors, with cystic regions found in BRCA1-KPC tumors. Murine pancreatic stellate cells from Mist1(KRasG12D/+) were alpha-SMA+ and produced IL-6 similar to human stellate cells. STAT3 was constitutively phosphorylated at Tyr705 in both malignant and stromal cells of the tumor microenvironment in each GEMM. Similar to patients with advanced PDA, high-throughput bioplex analysis revealed plasma from tumor-bearing mice contained high levels of IL-6 (mean 4729 pg/ml) and other important inflammatory factors. Time course data from the Mist1(KRasG12D/+) GEMM revealed elevated levels of MDSC at early stages of 3 months (4.6%±1.7), with higher levels (8.9%± 0.3) by 8 months as compared to healthy, age matched littermates (2.3%). Similarly, MDSC were significantly higher in BRCA1-KPC mice (14.2%±5.4; mean age 77 days) versus healthy, age-matched littermates (3.94%±0.3; mean age 67 days). These data demonstrate that multiple spontaneously arising GEMM for PDA display a phenotype comparable to the microenvironment of human patients. Future studies will take advantage of these properties and use these models for therapeutic evaluation of agents targeting the IL-6/JAK/STAT pathway within the tumor microenvironment.

